# Osteo Porosis

**Published:** 1891-07

**Authors:** V. G. Hunt


### COMMUNICATIONS.

### OSTEO POROSIS.

*Mr. Editor:*

The article from the pen of A. Jasme commenting on that of T. H. Berner seems to me so full of errors as to the pathology of osteo porosis as to be entirely misleading. He claims that the disease is of recent origin; that it does not admit of successful treatment; yet, before concluding his article he admits that a "quack" of his acquaintance does make cures, and makes a specialty of treating the disease.

Fifty years ago the disease was to be met with on nearly every large farm and was so well understood that any farmer could diagnose the disease, and many of them could so far cure it as to preserve the animal in useful, serviceable condition for years. It prevailed most in malarious districts, but was not regarded as contagious or infectious. In the great corn belt of the West where clover and timothy were not much used, and corn and corn fodder were the principal foods, the disease was very common. But in the same region to-day, a half-dozen cases cannot be found in a year's practice, and the ordinary, clod-hopping, country veterinarian finds no trouble in fixing up the majority of these. It is true there is a stage of the disease frequently reached before treatment, when every bone in the structure is diseased, and the tendons lose their attachments—when medication is useless. So there is a stage when a fire in a burning building is beyond the control of the firemen; but there was a stage when a bucket of water would have extinguished it.

The time was when many barbarous methods were resorted to in hope of curing the disease. But as the nature and causes of the ailment came to be understood, more rational and humane treatment was found efficatious. When it was found that the diet was largely responsible and a corresponding change was made, the cases of the disease began to decrease in number, and now when they do occur, are amenable to treatment.

In the February number of the *N. Y. Veterinary Review*, I gave my treatment which I have found to give the best results. So-called scientists may attempt to prove the disease contagious, infectious due to germs, &c., but the facts are wanting to establish any of their theories. Many of our scientific writers are about as well equipped for their business as the man I heard of some years ago. He was taking a ride over the Central road. He was gazing from the car windows on the varying landscape. A son of the soil occupied an adjoining seat. Our traveller, pointing to the fields, asked the farmer as they passed under review, what the vegetation was. Having answered a number of these queries, the farmer in turn became inquisitive and said: "You don't appear very familiar with country life. May I ask where you live and what business you are in?" To which came the reply: "No, I seldom get to the country; my business is very confining. I live in Chicago and am editor of an agricultural weekly."

If these theoretical veterinarians who write so learnedly on the diseases of stock would take a trip to the country occasionally, and come into contact once and awhile with an actual living, breathing horse or cow, instead of consorting only with the cows and horses of their imaginations, they might find there is more in heaven and earth than is dreamed of in their philosophies.

In such case, too, it might not be possible, as it is now, for a newly-fledged veterinarian to become an Assistant State Veterinarian, and go about the country shooting valuable horses for supposed glanders, and thus obliterate all evidence of ignorance and mistaken diagnosis. But purity and politics do not always walk arm in arm with knowledge, hence ignorant pretension with a political pull gets to the front while modest merit is relegated to obscurity.

Eastern legislation is tending to placing the entire live stock interest in the hands of a few. We, in the West, are watching for the outcome of your quarantine laws.

We do not take much stock in Court findings. I believe it was a New Jersey Court that granted a woman a divorce from her husband on account of his impotency, and the same Court convicted the husband of bastardy.

Good, wholesome laws for the protection of the live stock interests are desirable, but they should be framed so as not to be in the interest of the unprincipled money seeker. But little legislation is needed. Self interest is usually ample for protection. Contagious diseases will be speedily stamped out without legislation; as every stock owner is interested and ready to co-operate in effective measures.

I am truly glad that there are several journals in the country striving to elevate the standard of veterinary qualifications. True, practical knowledge is what is needed, not unsubstantial theories. Every stock owner should be a subscriber to some good stock journal. As for myself I would prefer to economize in anything rather than in my veterinary literature.

V. G. HUNT, V. S.

